# Traditional processing increases biological activities of *Dendrobium offificinale* Kimura et. Migo in Southeast Yunnan, China

**DOI:** 10.1038/s41598-022-17628-8

**Published:** 2022-08-31

**Authors:** Di Zhou, Ying Zhao, Zhilin Chen, Xiuxiang Yan, Yanqiang Zhao, Lu Gao, Lixin Yang

**Affiliations:** 1 Key Laboratory of Economic Plants and Biotechnology, Kunming Institute of Botany, Chinese Academy of Sciences, Kunming, 650201 Yunnan China; 2 Bio-Innovation Center of DR PLANT, Kunming Institute of Botany, Chinese Academy of Sciences, Kunming, 650201 Yunnan China; 3 Center of Biodiversity and Indigenous Knowledge, Kunming, 650034 Yunnan China; 4 College of Forestry and Vocational Technology in Yunnan, Kunming, 650224 Yunnan China; 5 School of Ethnic Medicine, Yunnan Minzu University, Kunming, 650504 Yunnan China; 6 College of Pharmacy, Guizhou University of Traditional Chinese Medicine, Guiyang, 550025 Guizhou China

**Keywords:** Ecology, Plant sciences

## Abstract

The orchid *Dendrobium officinale* grows throughout southeast China and southeast Asian countries and is used to treat inflammation and diabetes in traditional Chinese medicine. Tie pi feng dou is a well-known traditional Chinese medicine made from the dried *D. officinale* stems. Processing alters the physicochemical properties of TPFD; however, it is unclear how processing affects the quality and medicinal value of this plant. Here, we analyzed and compared the chemical composition of fresh stems of *D. officinale* and TPFD and explored possible explanations for the enhanced medicinal efficacy of processed *D. officinale* stems using qualitative and quantitative methods. To identify the components of FSD and TPFD, we used ultra-high-performance liquid chromatography combined with mass spectrometry in negative and positive ion modes and interpreted the data using the Human Metabolome Database and multivariate statistical analysis. We detected 23,709 peaks and identified 2352 metabolites; 370 of these metabolites were differentially abundant between FSD and TPFD (245 more abundant in TPFD than in FSD, and 125 less abundant), including organooxygen compounds, prenol lipids, flavonoids, carboxylic acids and their derivatives, and fatty acyls. Of these, 43 chemical markers clearly distinguished between FSD and TPFD samples, as confirmed using orthogonal partial least squares discriminant analysis. A pharmacological activity analysis showed that, compared with FSD, TPFD had significantly higher levels of some metabolites with anti-inflammatory activity, consistent with its use to treat inflammation. In addition to revealing the basis of the medicinal efficacy of TPFD, this study supports the benefits of the traditional usage of *D. officinale*.

## Introduction

The therapeutic properties of traditional Chinese medicines (TCMs) are derived from the collective contributions of their various chemical components, most of which are secondary metabolites and saccharides^1,2^. Plants used in TCM accumulate secondary metabolites, including polysaccharides, alkaloids, amino acids, flavonoids, phenols, coumarins, terpenoids, and benzyl compounds, which enable them to adapt to diverse environmental conditions. These metabolites possess various physiological activities that underpin their medicinal value when consumed. Before the clinical use of a TCM, the raw materials are typically subjected to traditional processing methods^3^, which change the physicochemical properties of the herbal materials. This can transform certain bioactive/toxic components, which is likely the primary way in which processing affects the therapeutic properties of TCM ingredients^4^.

The perennial epiphytic herb *Dendrobium officinale* Kimura & Migo belongs to the Orchidaceae family and is native to China and southeast Asian countries^5,6^. In TCM, this plant is used as a medicine or food to nourish “yin,” clearing heat, toning the stomach, and promoting fluid production^7^. For over 1000 years, this herb has been processed and used by ethnic groups in southwest Yunnan, China, to treat inflammation and diabetes^8^. According to the Pharmacopoeia of China (2020 edition), fresh stems of *D. officinale* (FSD) should be harvested, and the leaves and stem epidermis should be removed. The stem should then be semi-dried to a 45% moisture content before being twisted into a spring shape under heat. The resulting products, which have a moisture content of < 12%, are known as Xifengdou (translated as Tie pi feng dou, TPFD). Detailed records on the traditional processing of TPFD in China began in the Qing Dynasty^9^.

Characterizing the composition of TCMs such as TPFD can inform the development of medicines and supplements with similar properties and help establish quality control standards for these biological products, which vary in activity levels. Conventional chemical research into herbal materials involves the systematic isolation of their chemical constituents, followed by qualitative and quantitative comparisons, chemical profiling, and identification of chemical markers^10–13^. Metabolomics is widely used to elucidate the chemical compositions of herbal medicines^14^. Typically performed using liquid chromatography with mass spectrometry (LC–MS), metabolomics is a powerful approach for elucidating the global profiles of complex secondary metabolites by measuring their presence, abundance, and chemical structures^15,16^. LC–MS is also employed to identify marker compounds used to distinguish between raw and processed herbal medicines^17–19^.

TPFD is a well-known traditional product of *D. officinale*; however, the chemical composition of *D. officinale* is complex, and conventional analytical approaches are time-consuming and have not yielded a complete chemical characterization of the major differences between FSD and TPFD. Previous studies of the chemical composition of *D. officinale* have mainly focused on elucidating the botany, traditional use, phytochemistry, and pharmacology of *D. officinale*. While some studies have examined properties pertaining to the quality and safety of this herb^20^ and omics studies have explored the biosynthetic pathways and regulation mechanisms of the plant’s bioactive compounds^21^, the major chemical differences between raw and processed TPFD products are largely unknown. Exploring the chemical changes that occur during *D. officinale* processing will therefore provide helpful information for understanding the therapeutic characteristics of TPFD. Here, we analyzed the chemical constituents of TPFD and FSD and explored the effect of traditional processing on the therapeutic value of *D. officinale* using a non-targeted metabolomics method and a variety of chromatographic techniques. We then examined the differences between FSD and TPFD using multivariate and univariate statistical analyses to elucidate the main chemical transformations that occur during *D. officinale* processing.

## Materials and methods

### Regulatory statement

The plant experiments were performed in accordance with relevant guidelines and regulations.

### Reagents and materials

All chemicals and solvents used were of analytical or high-performance liquid chromatography grade. Water, acetonitrile, methanol, and formic acid were from Thermo Fisher Scientific (Waltham, MA, USA). L-2-chlorophenylalanine was obtained from Shanghai Hengchuang Biotechnology Co., Ltd. (Shanghai, China).

FSD and TPFD samples were obtained from the local market in Guangnan County, Wenshan Prefecture, Yunnan Province, southwest China. *D. officinale* is a National Geographic Indication Product. The raw materials for TPFD and FSD were collected from the fresh stems of *D. officinale* from the same batch in April 2021 and authenticated by Prof. Lixin Yang, Chinese Academy of Sciences. Type specimens were deposited in the Kunming Institute of Botany Herbarium (sample numbers: TPFD20210401 and FSD20210402).

### Sample preparation

Similarly sized samples were selected for analysis. Each sample was prepared in quadruplicate. First, all samples were thoroughly ground. For the analysis, 80 mg of FSD (Sample No.: DF-1-1 to DF-1-4) and TPFD (Sample No.: DF-F-1 to DF-F-4) was transferred into a 1.5-mL microfuge tube. Twenty microliters of internal standard (L-2-chlorophenylalanine, 0.3 mg/mL; methanol), 1 mL methanol–water (V:V = 7:3), and two small steel balls were added. The samples were chilled to − 20 °C for 2 min and then ground at 60 Hz for 2 min, extracted with ultrasonic waves for 30 min in an ice-water bath, and incubated at − 20 °C for 20 min. The samples were centrifuged at 4 °C and 13,000 rpm for 10 min. Then, a glass syringe was used to collect 150 μL of supernatant, which was filtered through microfilters (0.22 μm). The filtrate was transferred into LC vials, which were stored at − 80 °C until analysis.

For quality control (QC), pooled samples were prepared by mixing aliquots of all the samples.

### Secondary metabolite analysis

An ultra-high-performance liquid chromatography (UHPLC, Dionex Ultimate 3000 RS) with a mass spectrometer (Q-Exactive plus quadrupole-Orbitrap) equipped with a heated electrospray ionization (ESI) source (Thermo Fisher Scientific) was used to analyze the metabolic profiles in ESI positive and negative ion modes. The column (ACQUITY UPLC HSS T3, 1.8 μm, 2.1 × 100 mm) was employed in positive and negative modes. The elution reagents were (A) water with 0.1% (v/v) formic acid and (B) acetonitrile with 0.1% (v/v) formic acid, and the gradient was as follows: 0 min, 5% B; 1 min, 5% B; 2.5 min, 30% B; 6 min, 50% B; 7 min, 70% B; 10 min, 80% B; 12 min, 100% B; 14 min, 100% B; 14.2 min, 5% B; and 16 min, 5% B at 0.35 mL/min and a column temperature of 40 °C. Samples were maintained at 4 °C during analysis. The injection volume was 5 μL.

The mass range was detected between 100 and 1200 mass-to-charge ratio (m/z). A resolution of 70,000 was used for full MS scans, and 17,500 was used for higher-energy collisional dissociation (HCD) MS/MS scans, with a collision energy of 10, 20, and 40 eV. The mass spectrometer was operated as follows: spray voltage, 3800 V(+) and 3200 V(−); sheath gas flow rate, 40 arbitrary units; auxiliary gas flow rate, 15 arbitrary units; capillary temperature, 320 °C; auxiliary gas heater temperature, 350 °C; and S-lens RF level, 55. Every four samples, a QC sample was injected to assess repeatability.

### Statistical analysis

Progenesis QI V2.3 (Nonlinear Dynamics, Newcastle, UK) was used for baseline filtering, peak identification, integral retention time correction, peak alignment, and normalization of raw LC–MS data, with 5 ppm precursor tolerance, 10 ppm product tolerance, and 5% product ion threshold. Compound identification was based on comparing the precise m/z values, secondary fragments, and isotopic distribution with the Human Metabolome Database (HMDB) for qualitative analysis.

The data were further processed by removing peaks with missing values (ion intensity = 0) in more than 50% of groups by replacing zero values with half of the minimum value and by screening according to the qualitative results of the compound. Compounds with scores below 36 (of 60) were deemed to be inaccurate and removed.

A data matrix was generated from the positive and negative ion data and used for principal component analysis (PCA) in R. Orthogonal partial least squares discriminant analysis (OPLS-DA) and partial least squares discriminant analysis (PLS-DA) were used to identify the metabolites that differed between groups. Seven-fold cross-validation and 200 response permutation tests were used to prevent overfitting and evaluate the quality of the model.

Variable importance of projection (VIP) values from OPLS-DA were used to rank the contribution of each variable to the discrimination of groups. A two-tailed Student’s *t*-test was used to verify the significance of the differences in metabolite abundance between the groups. Metabolites with VIP > 1.0 and *P* < 0.05 were selected as differentially abundant.

## Results

### Identification of metabolite diversity in *D. officinale*

**Figure 1:**
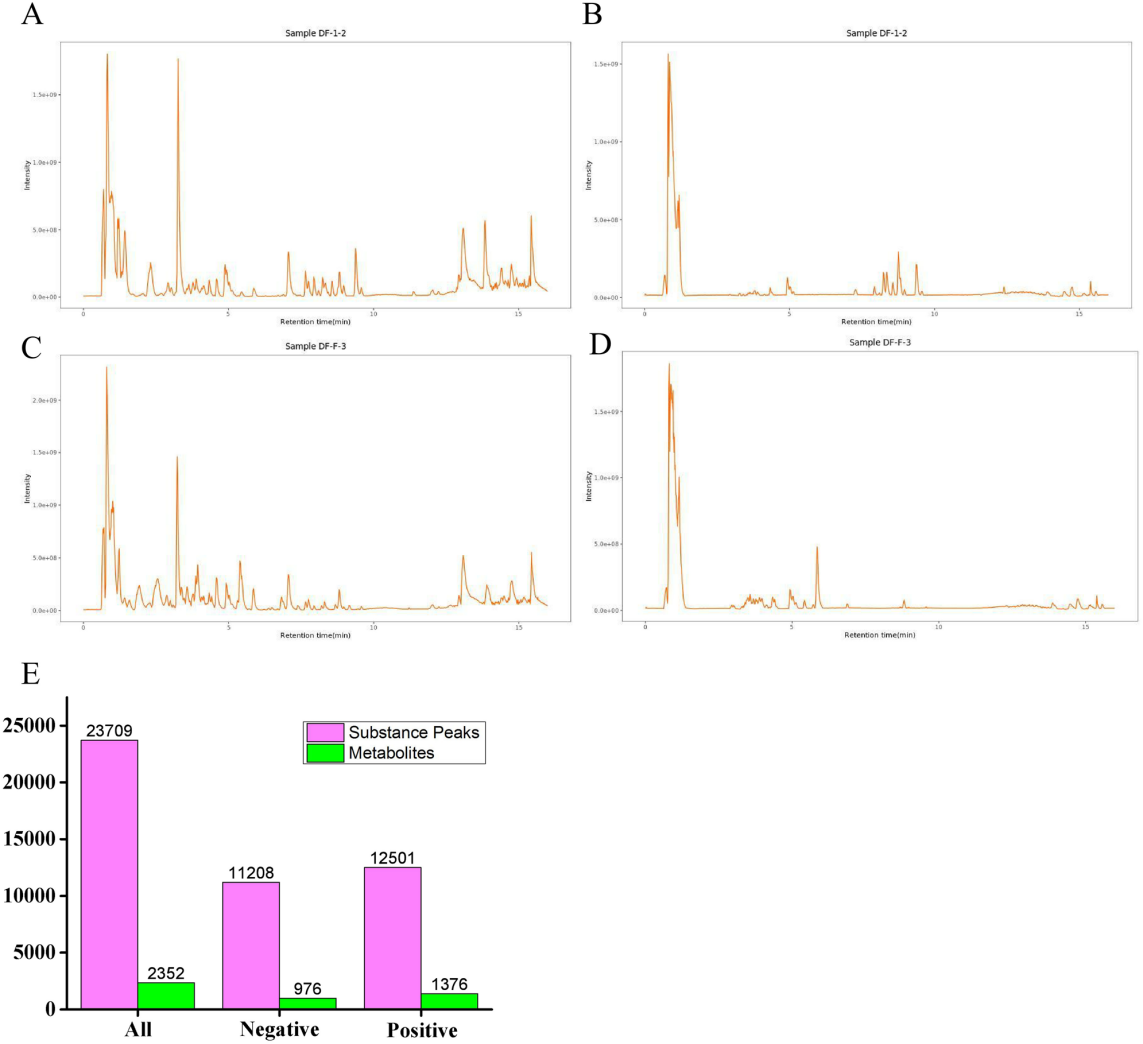
TICs of methanol extracts from FSD and TPFD samples and the global metabolites identified. (**A**): TICs of FSD samples (ESI+). (**B**): TICs of FSD samples (ESI–). (**C**): TICs of TPFD samples (ESI+). (**D**): TICs of TPFD samples (ESI–). E: Numbers of the substance peaks and identified metabolites.

Typical total ion current chromatograms (TICs) of FSD samples are presented in Fig. 1A (ESI+) and Fig. 1B (ESI−), and TICs of TPFD samples are presented in Fig. 1C (ESI+) and Fig. 1D (ESI−). A total of 23,709 substance peaks were detected in FSD and TPFD samples using the UHPLC Q-Exactive plus quadrupole-Orbitrap mass spectrometer, among which 2352 metabolites were identified (976 metabolites from the negative ion model (ESI−) and 1376 metabolites from the positive ion mode (ESI +) (Fig. 1E).

**Figure 2:**
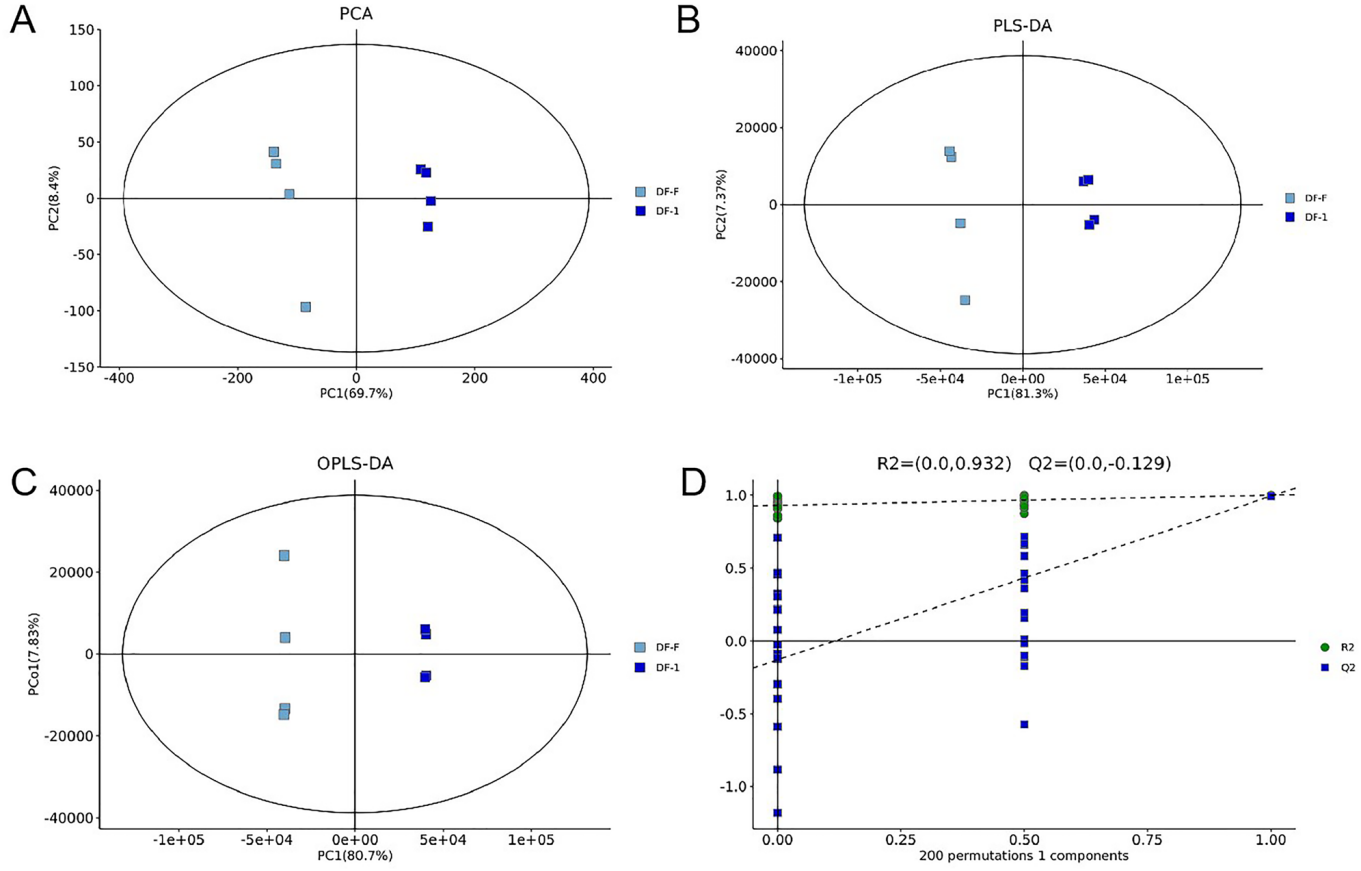
Model of the multivariate analysis and its cross-validation. (**A**): PCA for TPFD samples versus FSD samples. (**B**): PLS-DA for TPFD samples versus FSD samples. (**C**): OPLS-DA for TPFD samples versus FSD samples. (**D**): Response permutation testing of the model predicted by OPLS-DA.

FSD and TPFD samples contained all the identified metabolites, but the relative contents of individual compounds were remarkably different between the two groups (Fig. 2). However, metabolite contents were similar in the four biological replicates of an individual sample. The PCA plots between FSD and TPFD samples also showed clear differences (Fig. 2A); for example, PC1 was clearly separated between FSD and TPFD and represented 69.7% of the difference in their chemical compositions. Figure 2B presents the PLS-DA of the two groups. The R2 value of the PCA and Q value of the PLS-DA (Fig. 2) show that compound abundances between FSD and TPFD samples were statistically significantly different. OPLS-DA, another supervised method, was used to highlight the quantitative variation in the metabolites between TPFD and FSD samples (Fig. 2C). Cross-validation with 200 permutations supported the reliability of this OPLS-DA model, with R2 and Q2 intercepts of 0.932 and 0.129, respectively (Fig. 2D). These results show that TCM processing techniques lead to significant changes to the metabolite contents of *D. officinale*.

### Characterization of five categories of differentially abundant metabolites

**Figure 3:**
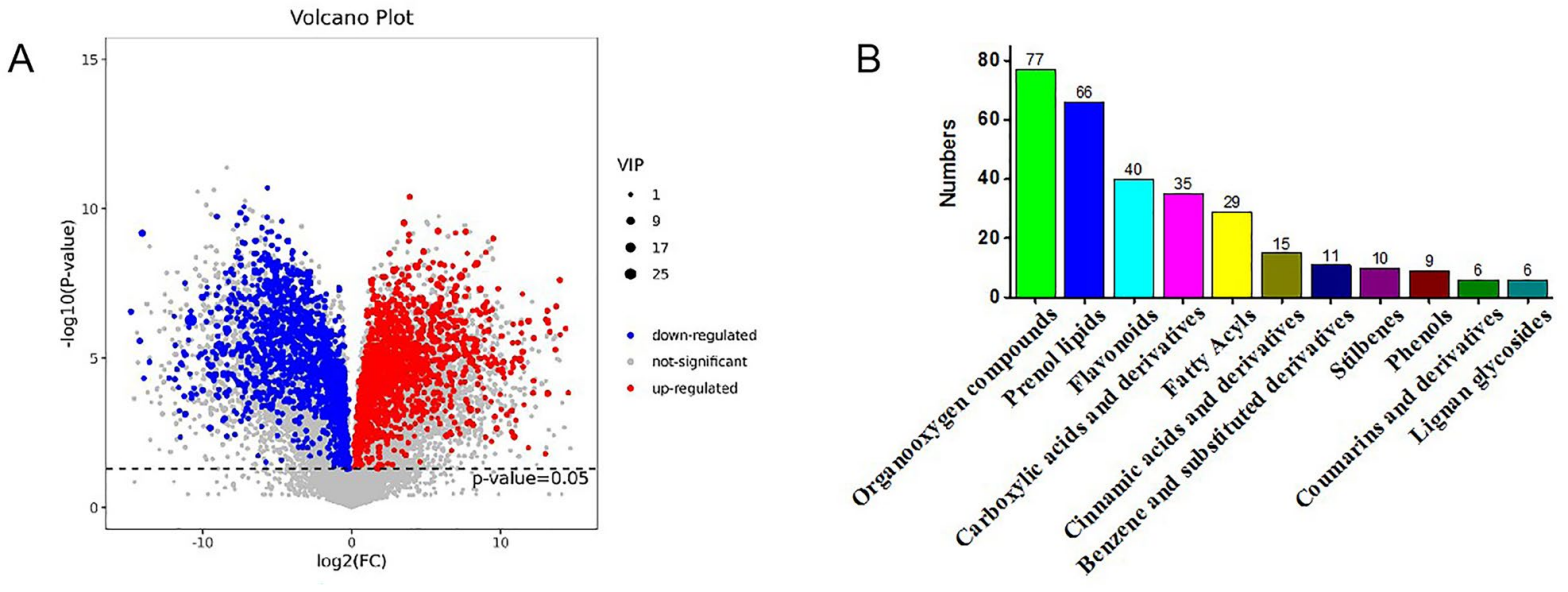
Differentially abundant metabolites between TPFD and FSD. (**A**): Volcano plot of the 2352 metabolites identified. (**B**): Main classes of differentially abundant metabolites.

Pairwise comparisons of metabolite abundances in FSD and TPFD using the OPLS-DA model identified differentially abundant metabolites based on the VIP value. Next, all identified and annotated metabolites were screened for different abundances between FSD and TPFD samples (Fig. 3A). Using the set criteria (VIP > 1; *P* < 0.05), 370 metabolites were found to be significantly differentially abundant between TPFD and FSD, the majority of which were organooxygen compounds, prenol lipids, flavonoids, carboxylic acids and their derivatives, and fatty acyls (Fig. 3B).

**Figure 4:**
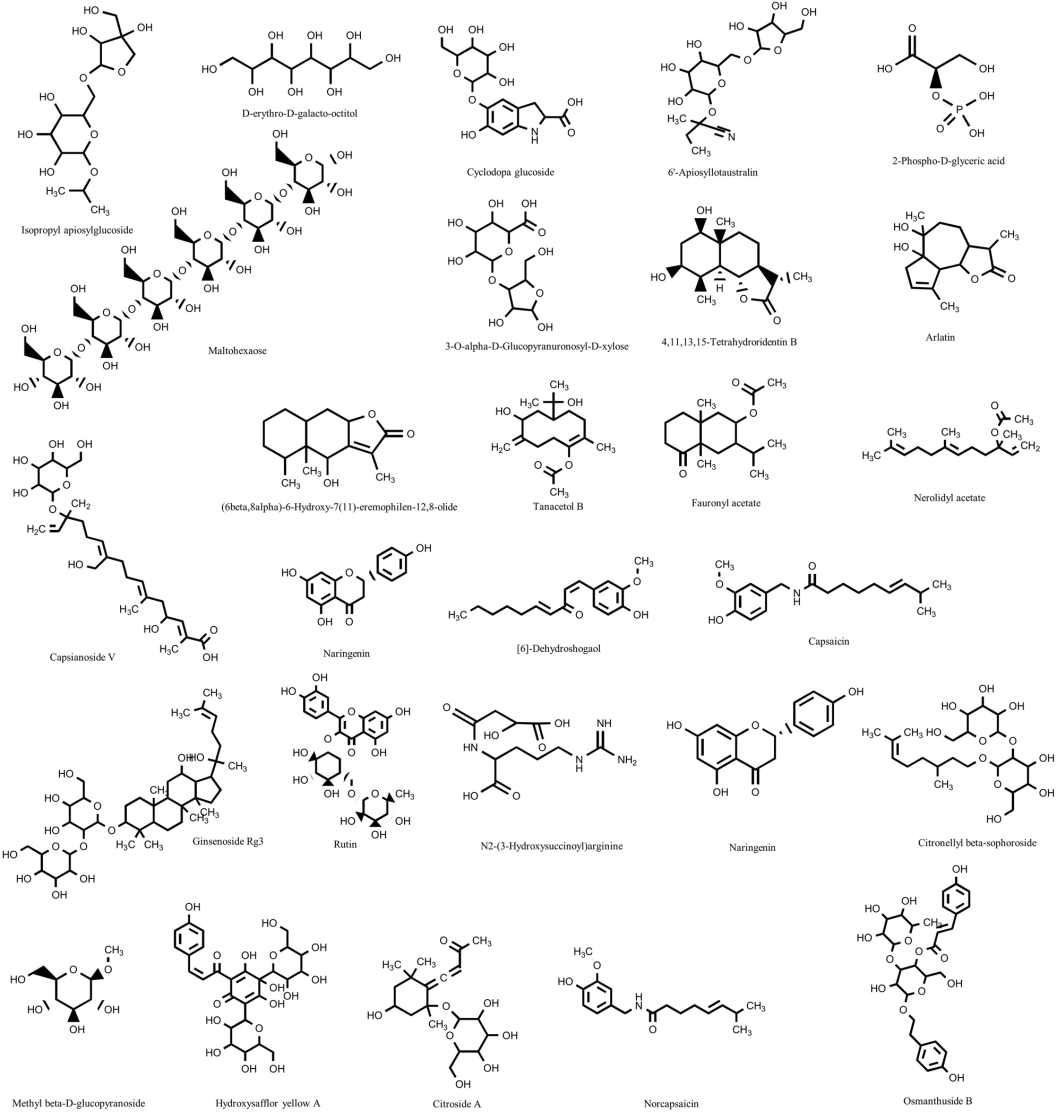
Chemical structures of typical compounds from TPFD.

First, organooxygen compounds were significantly more abundant in TPFD than in FSD samples. These compounds, especially carbohydrates and carbohydrate conjugates, directly contribute to the physiological activity of *D. officinale* products. Carbohydrates and carbohydrate aggregates from *D. officinale* have antioxidant, anti-tumor, immune-enhancing, and anti-inflammatory effects; they protect the liver and nerves; and they are useful for the treatment of diabetes and the intestinal microbiome^22^. A total of 77 organooxygen compounds were significantly differentially abundant between FSD and TPFD samples in this study, accounting for 20.8% of all the differentially abundant metabolites. Of these, 74 compounds were carbohydrates or carbohydrate aggregates. Sixty-one carbohydrates and carbohydrate aggregates were significantly more abundant in TPFD samples, while 13 carbohydrates and carbohydrate aggregates were significantly less abundant (Table 1). In particular, isopropyl apiosylglucoside, D-erythro-D-galacto-octitol, cyclodopa glucoside, 6'-apiosyllotaustralin, and maltohexaose (Fig. 4) were an average of 5.80 × 10^11^ times more abundant in TPFD samples than FSD, likely due to the TCM processing. By contrast, 2-phospho-D-glyceric acid and 3-*O*-alpha-*D*-glucopyranuronosyl-*D*-xylose (Fig. 4) were an average of 3.72 × 10^−12^ times less abundant in TPFD than in FSD. In addition, 44 carbohydrates and carbohydrate aggregates were at least twice as abundant in TPFD as in FSD, but only 9 compounds were half as abundant. These results suggest that TCM processing positively affects the accumulation of carbohydrates in TPFD.

**Table 1:** Information of organooxygen compounds with significant changes.

Metabolites	Compound ID	m/z	Retention time(min)	VIP	*P*-value	log2(FC)
Isopropyl apiosylglucoside	HMDB0041513	353.145	3.952	2.124	0.000	39.862
D-erythro-D-galacto-octitol	HMDB0029953	281.063	2.998	1.763	0.000	39.372
Cyclodopa glucoside	HMDB0029833	380.095	0.771	1.633	0.000	39.126
6'-Apiosyllotaustralin	HMDB0034207	416.155	1.184	1.190	0.000	38.195
Maltohexaose	HMDB0012253	1013.316	1.004	1.108	0.000	38.010
N-(1-Deoxy-1-fructosyl)alanine	HMDB0038662	234.097	0.816	1.797	0.000	15.901
(E)-2-O-Cinnamoyl-beta-D-glucopyranose	HMDB0035880	328.139	0.867	2.119	0.000	13.310
Tetraphyllin B	HMDB0029914	310.090	2.009	2.573	0.000	10.850
Linamarin	HMDB0033699	230.102	1.268	2.371	0.000	10.061
Ethylvanillin glucoside	HMDB0037682	351.105	4.321	2.626	0.000	7.328
Galactopinitol B	HMDB0035321	395.095	0.850	2.004	0.000	6.840
D-Sedoheptulose 7-phosphate	HMDB0001068	291.047	0.867	1.079	0.000	6.675
3-Furanmethanol glucoside	HMDB0032924	305.088	1.454	1.152	0.003	6.641
Ethyl beta-D-glucopyranoside	HMDB0029968	231.084	1.385	1.657	0.000	6.401
Furaneol 4-glucoside	HMDB0032992	308.134	0.816	1.709	0.000	6.283
Trans-p-Coumaric acid 4-glucoside	HMDB0039509	349.090	3.424	2.001	0.000	4.798
Glucosamine	HMDB0001514	162.076	0.833	1.362	0.000	4.542
Trans-o-Coumaric acid 2-glucoside	HMDB0033581	307.082	3.888	1.036	0.002	4.266
Casuarine 6-alpha-D-glucoside	HMDB0031999	348.130	3.438	1.023	0.000	4.193
Citrusin C	HMDB0038708	307.119	6.226	1.522	0.000	4.156
Salviaflaside	HMDB0033705	521.130	4.254	1.106	0.001	4.017
Trehalose 6-phosphate	HMDB0001124	423.090	0.850	3.331	0.000	3.947
4-O-Methylgalactinol	HMDB0033558	379.121	1.184	1.649	0.000	3.791
2-(3-Hydroxyphenyl)ethanol 1'-glucoside	HMDB0038332	345. 119	3.245	1.815	0.000	3.736
Chlorogenic acid	HMDB0003164	377.084	0.867	1.422	0.000	3.544
Methyl beta-D-glucopyranoside	HMDB0029965	217.068	1.184	5.375	0.000	3.525
Dihydromelilotoside	HMDB0038334	351. 105	3.652	1.355	0.000	3.474
Sphalleroside A	HMDB0032767	365. 120	4. 194	1.335	0.000	3.326
Moringyne	HMDB0031724	357.119	3.481	2.348	0.000	3.118
Phosphoribosylformylglycineamidine	HMDB0000999	331.100	1.184	1.468	0.000	3.066
Di-O-methylcrenatin	HMDB0032742	391.125	3.524	2.012	0.000	2.973
Xylobiose	HMDB0029894	305.084	1.184	1.268	0.000	2.890
Hydroxytyrosol 1-O-glucoside	HMDB0041024	339.105	3.694	1.968	0.000	2.671
5a,6a-Epoxy-7E-megastigmene-3a,9e-diol 3-glucoside	HMDB0031676	433.208	3.674	4.134	0.000	2.426
Foeniculoside VIII	HMDB0033009	371.168	3.507	4.373	0.001	2.381
Coniferin	HMDB0013682	341.124	3.803	1.181	0.000	2.216
Benzyl gentiobioside	HMDB0041515	455.152	3.342	3.494	0.000	2.071
N-Acetyl-D-glucosamine	HMDB0000215	204.087	0.833	2.130	0.000	2.061
5-Aminoimidazole ribonucleotide	HMDB0001235	313.092	4.004	1.806	0.000	2.029
2'-Methoxy-3-(2,4-dihydroxyphenyl)-1,2-propanediol 4'-glucoside	HMDB0039473	405.140	2.742	1.284	0.000	1.961
1-(3,4-Dimethoxyphenyl)-1,2-ethanediol 2-O-b-D-glucoside	HMDB0034627	383.131	3.246	3.610	0.000	1.943
Chlorogenoquinone	HMDB0029383	375.069	0.867	1.008	0.000	1.694
Gentiotriose	HMDB0029910	549.167	0.810	3.078	0.000	1.663
Ptelatoside A	HMDB0032600	827.299	3.952	1.095	0.000	1.566
beta-D-Galactopyranosyl-( 1− > 3)-beta-D-galactopyranosyl-(1− > 6)-D-galactose	HMDB0038853	527.158	1.004	5.111	0.000	1.442
Salicin	HMDB0003546	287.110	2.183	1.423	0.000	1.432
Mannan	HMDB0029931	705.185	0.799	1.223	0.001	1.409
6-Kestose	HMDB0033673	543.132	0.816	4.641	0.000	1.289
(S)-alpha-Terpinyl glucoside	HMDB0029856	361.187	5.499	1.677	0.001	1.196
5-Hydroxymethyl-2-furancarboxaldehyde	HMDB0034355	127.039	0.850	2.361	0.000	1.153
Kelampayoside A	HMDB0038714	477.161	3.524	1.270	0.000	1.131
Linalool 3,6-oxide primeveroside	HMDB0035489	463.219	3.845	2.555	0.000	1.129
Pseudouridine 5'-phosphate	HMDB0001271	305.018	1.197	1.079	0.000	0.955
Methyl salicylate O-[rhamnosyl-(1− > 6)-glucoside]	HMDB0033138	459.151	4.405	3.121	0.002	0.938
Vanilloloside	HMDB0032013	631.225	3.417	1.043	0.000	0.918
Linalool oxide D 3-[apiosyl-(1− > 6)-glucoside]	HMDB0031367	509.224	3.824	4.502	0.000	0.881
Verbasoside	HMDB0039233	461.167	3.417	1.008	0.000	0.853
Linalool 3,7-oxide beta-primeveroside	HMDB0036571	482.259	4.067	1.773	0.000	0.766
Benzyl beta-primeveroside	HMDB0041190	401.145	3.632	1.550	0.002	0.757
Myzodendrone	HMDB0041273	387.130	3.310	1.292	0.001	0.640
trans-p-Menthane-1,7,8-triol 8-glucoside	HMDB0034784	373.183	3.403	4.657	0.003	0.628
Pteroside P	HMDB0036608	441.176	3.931	1.233	0.007	0.401
2-O-beta-D-Glucopyranuronosyl-D-mannose	HMDB0039722	337.078	0.860	1.176	0.007	0.384
Trehalose	HMDB0000975	387.114	0.804	7.692	0.004	0.301
4-O-beta-D-Galactopyranosyl-D-xylose	HMDB0038864	357.104	0.804	1.063	0.032	− 0.245
3,5-Dihydroxyphenyl-1-O-(6-O-galloyl-beta-D-glucopyranoside)	HMDB0039307	439.086	0.810	8.940	0.000	− 0.709
a-L-Arabinofuranosyl-(1− > 3)-[a-L-arabinofuranosyl-(1r5)]-L-arabinose	HMDB0041223	432.171	0.816	2.132	0.000	− 0.894
3,4,5-Trimethoxyphenyl 2,6-digalloylglucoside	HMDB0039312	631.128	3.696	1.692	0.000	− 0.921
(S)-Nerolidol 3-O-[a-L-Rhamnopyranosyl-(1− > 4)-a-L-rhamnopyranosyl-(1− > 2)-b-D-glucopyranoside]	HMDB0040845	721.366	8.316	5.223	0.000	− 1.917
(S)-Nerolidol 3-O-[a-L-rhamnopyranosyl-(1− > 4)-a-L-rhamnopyranosyl-(1− > 6)-b-D-glucopyranoside]	HMDB0040846	699.356	8.331	6.236	0.000	− 2.019
cis-p-Coumaric acid 4-[apiosyl-(1− > 2)-glucoside]	HMDB0037088	481.132	4.215	1.852	0.000	− 2.756
Gluconasturtiin	HMDB0038423	441.100	3.342	2.914	0.000	− 3.586
Benzyl O-[arabinofuranosyl-(1− > 6)-glucoside]	HMDB0041514	383.135	4.904	1.221	0.000	− 3.883
6-Phosphogluconic acid	HMDB0001316	277.032	0.850	2.730	0.000	− 4.135
Mangalkanyl glucoside	HMDB0036015	431.265	7.825	2.676	0.000	− 9.586
3-O-alpha-D-Glucopyranuronosyl-D-xylose	HMDB0039723	651.161	8.771	1.070	0.000	− 37.900
2-Phospho-D-glyceric acid	HMDB0003391	230.991	0.916	1.128	0.000	− 38.044

Second, Prenol lipids are naturally occurring and are formed by the condensation of isoprene subunits^23^. Prenol lipids have critical roles not only as structural components of cell membranes, but also as essential signaling molecules^24^; for example, vitamin K plays a key role in bone health and cardiovascular homeostasis^25^. Similarly, vitamins E and A, as well as ubiquinones, have crucial effects on the progression of age-related diseases and chronic conditions such as inflammation and diabetes^26^.

In this study, the number of differentially abundant prenol lipids in TPFD samples was second only to that of organooxygen compounds, accounting for 17.8% of all the differentially abundant metabolites. The relative concentrations of 46 prenol lipids were significantly higher in TPFD than in FSD samples, while 13 prenol lipids were significantly lower (Table 2). When compared with FSD samples, capsianoside V, 4,11,13,15-tetrahydroridentin B, arlatin, and (6beta,8alpha)-6-hydroxy-7(11)-eremophilen-12,8-olide (Fig. 4) were more abundant in TPFD samples, while ginsenoside Rg3, tanacetol B, fauronyl acetate, and nerolidyl acetate were less abundant (Fig. 4). The variation in these compounds indicates their different contributions to the final quality of TPFD products.

**Table 2:** Information of prenol lipids with significant changes.

Metabolites	Compound ID	m/z	Retention time (min)	VIP	*P*-value	log2(FC)
Capsianoside V	HMDB0030737	559.276	4.622	1.176	0.000	10.228
4,11,13,15-Tetrahydroridentin B	HMDB0036150	269.175	4.215	1.530	0.000	8.906
Arlatin	HMDB0035740	289.141	5.365	5.270	0.000	8.883
(6beta,8alpha)-6-Hydroxy-7(11)-eremophilen-12,8-olide	HMDB0035148	251.164	3.569	1.558	0.000	6.910
Artemin	HMDB0034696	249.148	4.278	1.250	0.000	5.738
Geniposidic acid	HMDB0034942	397.111	3.486	2.191	0.000	5.418
Lactaronecatorin A	HMDB0037529	233.154	4.215	1.404	0.000	5.401
4-Epiisoinuviscolide	HMDB0031378	249.148	3.859	1.018	0.000	5.304
Neryl rhamnosyl-glucoside	HMDB0029349	443.229	6.172	1.723	0.000	4.670
Epioxylubimin	HMDB0035613	253.180	3.941	2.115	0.000	4.607
(4R,5S,7R,11S)-11,12-Dihydroxy-1(10)-spirovetiven-2-one 11-glucoside	HMDB0033150	459.224	4.125	4.807	0.000	4.462
Abscisic alcohol 11-glucoside	HMDB0039636	457.208	4.125	2.120	0.000	4.350
Glucosyl passiflorate	HMDB0038141	679.402	5.876	1.176	0.000	4.204
Cinncassiol D1 glucoside	HMDB0034677	515.283	4.596	1.694	0.000	4.174
1,2-Dehydro-alpha-cyperone	HMDB0036589	217.159	3.818	2.495	0.000	4.124
Lucidenic acid D1	HMDB0038199	451.211	4.319	1.274	0.000	3.879
3,11,12-Trihydroxy-1(10)-spirovetiven-2-one	HMDB0038154	269.175	5.620	1.341	0.000	3.854
Citronellyl beta-sophoroside	HMDB0032839	461.240	4.341	8.101	0.000	3.534
Ipomeatetrahydrofuran	HMDB0040904	279.193	5.855	1.128	0.000	3.518
6alpha-Carissanol	HMDB0035309	235.169	4.131	3.475	0.000	3.244
Curcumol	HMDB0038122	219.174	4.342	3.389	0.000	3.010
Tsugarioside B	HMDB0035509	639.424	13.205	1.624	0.000	2.896
Rotundone	HMDB0036443	201.164	4.342	2.284	0.000	2.765
Aubergenone	HMDB0035827	237.185	4.004	1.933	0.000	2.534
Lubiminol	HMDB0029604	277.177	6.239	1.611	0.000	2.489
Crocin 3	HMDB0039121	675.262	4.236	2.972	0.000	2.227
Kiwiionoside	HMDB0038691	429.210	2.998	4.578	0.000	2.217
Icariside B8	HMDB0036846	411.199	3.673	4.161	0.000	1.975
Deoxyloganic acid	HMDB0037028	405.140	3.224	1.436	0.000	1.900
Oleoside 11-methyl ester	HMDB0041550	385.114	3.546	2.176	0.000	1.861
Pokeberrygenin	HMDB0034653	517.350	10.367	1.030	0.010	1.797
Citroside A	HMDB0030370	409.183	3.673	4.761	0.000	1.723
2beta,9xi-Dihydroxy-8-oxo-1(10),4,11(13)-germacratrien-12,6alpha-olide	HMDB0036662	261.112	5.408	1.062	0.046	1.659
Pisumionoside	HMDB0039947	443.168	5.216	2.374	0.000	1.626
Kenposide B	HMDB0039749	429.213	4.059	1.304	0.000	1.599
Ganoderenic acid D	HMDB0036059	557.273	9.555	1.126	0.000	1.528
6Z-8-Hydroxygeraniol 8-O-glucoside	HMDB0035025	377.182	3.888	5.028	0.000	1.480
Alpha-Cyperol	HMDB0035026	203.180	5.834	1.011	0.000	1.399
Oleoside dimethyl ester	HMDB0031350	417.140	3.567	4.651	0.000	1.346
Ganoderic acid H	HMDB0035987	595.285	10.581	1.731	0.000	1.134
(1S,4S)-Dihydrocarvone	HMDB0036080	135.117	3.901	1.232	0.000	1.043
Tricrocin	HMDB0002376	853.289	5.195	1.370	0.000	1.000
Nigroxanthin	HMDB0037122	567.420	14.345	3.147	0.001	0.741
Musabalbisiane C	HMDB0038682	607.215	5.642	1.126	0.003	0.590
(−)-trans-Carveol	HMDB0003450	153.127	3.631	1.014	0.000	0.490
Capsanthin 3,6-epoxide	HMDB0033260	583.414	14.345	2.24	0.016	0.315
Acuminoside	HMDB0029347	493.229	4.926	4.358	0.031	− 0.294
Ganoderic acid C1	HMDB0035627	1027.581	15.213	1.079	0.000	− 0.538
Veranisatin A	HMDB0040663	365.121	3.321	1.405	0.001	− 0.568
Rosmic acid	HMDB0032082	413.157	6.856	1.128	0.000	− 0.912
Gibberellin A37 glucosyl ester	HMDB0038611	509.236	6.154	1.844	0.001	− 0.932
Fasciculol C	HMDB0035853	531.366	13.395	1.367	0.002	− 0.973
S-Japonin	HMDB0035802	381.174	13.149	1.300	0.000	− 1.076
Geranylcitronellol	HMDB0032147	310.310	13.331	2.037	0.020	− 1.156
Ganoderic acid alpha	HMDB0033024	597.302	9.517	1.168	0.000	− 1.363
Cinncassiol A 19-glucoside	HMDB0035165	527.247	4.959	1.819	0.000	− 1.730
Neoxanthin	HMDB0003020	583.414	9.033	1.216	0.000	− 2.808
Fasciculic acid B	HMDB0036438	619.417	13.480	2.131	0.000	− 2.966
Stachyoside A	HMDB0039092	565.155	4.067	2.222	0.000	− 4.264
Boviquinone 4	HMDB0030057	435.251	10.624	3.230	0.000	− 5.012
Ginsenoside Rh3	HMDB0039645	622.467	12.443	1.336	0.000	− 5.823
Ganoderic acid eta	HMDB0036309	555.292	9.369	1.412	0.000	− 5.960
Nerolidyl acetate	HMDB0039630	309.207	7.925	4.241	0.000	− 7.130
Fauronyl acetate	HMDB0036422	325.202	7.669	2.714	0.000	− 7.408
Tanacetol B	HMDB0035075	341.197	5.341	1.279	0.001	− 11.550
Ginsenoside Rg3	HMDB0039546	823.460	8.331	1.239	0.000	− 38.364

Third, fruits and vegetables contain abundant quantities of flavonoids, which contribute to plant color and protect against microbial infection^27^. The properties of flavonoids depend on the arrangement of hydroxyl, methoxy, and glycosidic side groups and the conjugation between the A- and B-rings^28^. A total of 40 flavonoids, including common flavonoids such as naringin and rutin (Fig. 4), were significantly differentially abundant between TPFD and FSD samples (Table 3). After TCM processing, 26 flavonoids were significantly more abundant in TPFD than in FSD samples, while 14 flavonoids were significantly less abundant. Flavonoids possess anti-inflammatory and antioxidant activities and are considered potential therapeutic agents^28,29^. The content differences of these flavonoids therefore may influence the therapeutic characteristics of TPFD.

**Table 3:** Information of flavonoids with significant changes.

Metabolites	Compound ID	m/z	Retention time (min)	VIP	*P*-value	log2(FC)
Sideritiflavone	HMDB0038356	378.117	3.166	1.042	0.000	9.793
Luteolin 6-C-glucoside 8-C-arabinoside	HMDB0029258	633.142	3.673	1.188	0.000	8.126
Crosatoside A	HMDB0039124	647.158	3.859	1.868	0.000	5.321
6,8-Diglucosyldiosmetin	HMDB0037410	669.168	3.845	1.101	0.000	5.232
Pasternoside	HMDB0037743	625.176	3.859	3.995	0.000	4.922
Rhoifolin	HMDB0038848	623.162	3.867	2.865	0.000	4.717
4',5,8-Trihydroxyflavanone	HMDB0031824	271.061	5.858	10.133	0.000	4.266
Naringenin	HMDB0002670	273.076	5.855	5.399	0.000	4.219
5,7-Dihydroxyflavone	HMDB0036619	299.056	7.669	1.336	0.001	3.498
6-Glucopyranosylprocyanidin B2	HMDB0037403	741.203	4.321	1.282	0.001	3.440
Catechin 3',5-diglucoside	HMDB0037951	637.175	3.548	1.223	0.000	3.379
Norartocarpanone	HMDB0037314	287.056	5.149	1.353	0.001	3.061
Apigenin 7-[galactosyl-(1− > 4)-mannoside]	HMDB0037852	593.151	3.760	5.394	0.000	3.037
Kaempferol 3-[2''-(p-coumaroylglucosyl)rhamnoside]	HMDB0040538	739.188	4.341	1.204	0.001	2.804
Spinosin C	HMDB0037463	799.210	4.103	1.642	0.001	2.739
5,7-Dihydroxy-4'-methoxy-8-methylflavanone	HMDB0041321	301.107	6.411	1.245	0.000	2.609
(2R)-6,8-Diglucopyranosyl-4',5,7-trihydroxyflavanone	HMDB0037407	641.172	3.546	1.623	0.000	2.475
8-Hydroxyhesperetin 7-[6-acetylglucosyl-(1− > 2)-glucoside]	HMDB0041232	665.169	3.548	1.572	0.000	2.296
Kaempferol 3-(2''-rhamnosyl-6''-acetylgalactoside) 7-rhamnoside	HMDB0040541	763.210	3.952	1.130	0.000	2.295
Rutin	HMDB0003249	609.146	3.867	3.059	0.002	2.229
Dihydroprudomenin	HMDB0039357	539.138	0.804	3.411	0.000	1.990
KB 2	HMDB0033666	477.152	6.261	1.185	0.035	1.848
Isovitexin 2'–O–(6'''-feruloyl)glucoside	HMDB0038042	769.198	4.211	2.048	0.001	1.747
Scoparin 2''-xyloside	HMDB0038814	639.154	3.782	1.662	0.000	1.585
3',4',5'-Trimethoxyflavone	HMDB0033639	351.069	0.833	1.907	0.000	1.140
Isowertin 2''-rhamnoside	HMDB0037417	575.174	3.714	1.090	0.002	0.355
Kaempferol 3-arabinofuranoside 7-rhamnofuranoside	HMDB0037575	563.141	3.696	2.389	0.022	− 0.446
Aromadendrin 3,7-diglucoside	HMDB0040559	593.151	3.524	1.675	0.006	− 0.529
Myricetin 3-galactoside	HMDB0034358	959.182	1.044	1.678	0.001	− 0.550
2'',6''-Diacetylorientin	HMDB0038777	533.127	3.983	1.330	0.000	− 0.554
Apigenin 6-C-glucoside 8-C-arabinoside	HMDB0029260	565.155	3.714	3.660	0.001	− 0.613
Acacetin 7-[apiosyl(1− > 6)-glucoside]	HMDB0035023	579.170	3.797	3.713	0.000	− 0.715
3,6,7-Trihydroxy-4'-methoxyflavone 7-rhamnoside	HMDB0041455	469.111	6.389	1.059	0.004	− 0.742
Graveobioside B	HMDB0037454	595.166	3.528	2.773	0.000	− 0.874
Kaempferol 3-rhamnoside 7-xyloside	HMDB0039319	563.140	4.059	1.835	0.001	− 2.690
Chrysin 7-[rhamnosyl-(1− > 4)-glucoside]	HMDB0039934	563.175	4.575	1.128	0.019	− 2.797
Prunin 6''-O-gallate	HMDB0037582	587.137	4.046	1.555	0.000	− 5.046
Cyanidin 3-(6-caffeoylglucoside) 5-glucoside	HMDB0037983	754.177	5.454	1.328	0.000	− 5.927
6''-Malonylapiin	HMDB0037601	631.128	4.059	1.210	0.000	− 6.041
Licorice glycoside E	HMDB0031996	711.240	12.997	1.429	0.000	− 38.731

Finally, carboxylic acids, their derivatives, and fatty acyls are also major categories of compounds that are differentially abundant between TPFD and FSD samples. Here, 35 carboxylic acids and derivatives and 29 fatty acids were significantly differentially abundant between the samples (Tables 4 and 5). Carboxylic acids and derivatives are important substances in animal and plant metabolism and are used commercially in the synthesis of pesticides, herbicides, and insect repellents. Mounting evidence suggests that carboxylic acids and derivatives also have considerable pharmacological activities; for example, pentacyclic triterpenoid carboxylic acids have strong antioxidant, anti-inflammatory, antibacterial, anti-diabetic, and anti-tumor activities^30–32^. TPFD is traditionally used for the treatment of diabetes, cancer, and inflammation, among other conditions, suggesting that changes in carboxylic acids and their derivatives may determine the efficacy of TPFD. Short-chain fatty acids contribute to the flavor of *D. officinale*, and long-chain fatty acids can be degraded and transformed into various active flavor components through oxidation reactions^33^. The content difference of these fatty acids may therefore affect the flavor characteristics of TPFD.

**Table 4:** Information of carboxylic acids and derivatives with significant changes.

Metabolites	Compound ID	m/z	Retention time (min)	VIP	*P*-value	log2(FC)
N-Carboxyacetyl-D-phenylalanine	HMDB0039102	232.061	4.016	1. 111	0.000	37.990
Agaritinal	HMDB0040694	283. 140	1.704	1.536	0.000	14.545
D-1-[(3-Carboxypropyl)amino]-1-deoxyfructose	HMDB0038663	266. 123	0.816	8.636	0.000	11.310
(S)-2,3,4,5-Tetrahydropiperidine-2-carboxylate	HMDB0012130	110.060	0.850	1.034	0.000	7.307
N5-Acetyl-N2-gamma-L-glutamyl-L-ornithine	HMDB0039423	605.282	4.081	3.591	0.000	7. 153
Ustiloxin D	HMDB0041054	475.219	4.470	2. 133	0.000	6.074
N2-(3-Hydroxysuccinoyl)arginine	HMDB0032765	329.084	1. 184	4.284	0.000	5.518
N-Acetyl-L-glutamate 5-semialdehyde	HMDB0006488	156.066	0.906	1.428	0.000	2.683
2-Aminoheptanedioic acid	HMDB0034252	176.092	0.799	1.480	0.000	2.569
L-gamma-Glutamyl-S-allylthio-L-cysteine	HMDB0038515	367.065	2. 154	1.080	0.000	2.014
N2-Galacturonyl-L-lysine	HMDB0033105	305. 135	0.816	1. 179	0.001	1.973
Pyroglutamic acid	HMDB0000267	130.050	1. 184	1.414	0.000	1.853
N,N'-Bis(gamma-glutamyl)cystine	HMDB0038458	499. 114	0.886	1.916	0.004	− 0.407
N-gamma-Glutamyl-S-allylcysteine	HMDB0031874	579. 178	0.810	1.371	0.000	− 0.509
Isocitric acid	HMDB0000193	191.019	0.865	4.588	0.017	− 0.548
D-threo-Isocitric acid	HMDB0001874	191.019	1. 197	4.778	0.008	− 0.831
L-Valine	HMDB0000883	118.086	0.867	1.908	0.000	− 0.929
O-Acetylserine	HMDB0003011	130.050	0.867	1.791	0.000	− 1.471
L-Threonine	HMDB0000167	120.066	0.816	1.473	0.000	− 0.565
Citric acid	HMDB0000094	215.016	1. 184	8.410	0.000	− 1.718
(−)-Dioxibrassinin	HMDB0038634	306.997	1. 133	1.857	0.000	− 0.901
L-Proline	HMDB0000162	116.071	0.833	2.284	0.000	− 2.279
Glycylglycylglycine	HMDB0029419	377. 143	11.608	1.872	0.000	− 2.579
N6-Acetyl-5S-hydroxy-L-lysine	HMDB0033891	205. 118	0.850	1.225	0.000	− 2.623
(S)-2-Azetidinecarboxylic acid	HMDB0029615	140.011	0.906	1.057	0.000	− 2.920
L-2-Amino-3-(1-pyrazolyl)propanoic acid	HMDB0034267	156.077	0.721	1.572	0.000	− 2.960
L-Isoleucine	HMDB0000172	132. 102	1.422	8.430	0.000	− 2.970
Nigellimine N-oxide	HMDB0033436	237. 123	3.226	1.033	0.000	− 3. 139
L-Dihydroorotic acid	HMDB0003349	176.066	1.433	2.001	0.000	− 3.889
N5-(4-Methoxybenzyl)glutamine	HMDB0033598	267. 134	3.226	1.254	0.000	− 4.033
L-Glutamine	HMDB0000641	147.076	0.799	3.313	0.000	− 4.087
Pipecolic acid	HMDB0000070	147. 113	0.709	2.144	0.000	− 4. 142
N-Feruloylaspartic acid	HMDB0040830	327. 120	7.595	1.316	0.004	− 4.986
(2S,2'S)-Pyrosaccharopine	HMDB0038676	276. 155	2.289	1.017	0.000	− 7.770
L-2-Amino-5-hydroxypentanoic acid	HMDB0031658	172.037	1.683	1.822	0.006	− 15.423

**Table 5:** Information of fatty acyls with significant changes.

Metabolites	Compound ID	m/z	Retention time (min)	VIP	*P*-value	log2(FC)
1-(3-Methyl-2-butenoyl)-6-apiosylglucose	HMDB0039952	417. 137	4.173	3.630	0.000	6.660
3-Hydroxymethylglutaric acid	HMDB0000355	323.098	1.283	1.028	0.001	5.356
(3S,7E,9R)-4,7-Megastigmadiene-3,9-diol 9-[apiosyl-( 1− > 6)-glucoside]	HMDB0029766	549.255	3.696	1.583	0.000	4.962
Eriojaposide B	HMDB0038029	499.252	4.533	1.946	0.000	4.694
(3S,7E,9S)-9-Hydroxy-4,7-megastigmadien-3-one 9-glucoside	HMDB0036822	415. 198	4.298	2. 168	0.000	4.055
9-Hydroxy-7-megastigmen-3-one glucoside	HMDB0040701	417.213	4.059	1.208	0.000	2.952
3-Hydroxy-4,6-heptadiyne- 1-yl 1-glucoside	HMDB0038964	309.094	3.589	2.309	0.000	2.878
Blumenol C glucoside	HMDB0040668	395.204	4.321	2.859	0.000	2.553
Eriojaposide A	HMDB0038028	501.234	4.622	2.504	0.000	2. 171
(3S,5R,6S,7E,9x)-7-Megastigmene-3,6,9-triol 9-glucoside	HMDB0041176	435.224	3.696	1.929	0.000	2. 102
Isopentyl gentiobioside	HMDB0041512	393. 177	3.503	1.888	0.001	2.010
13-Oxo-9, 11-tridecadienoic acid	HMDB0034564	207. 138	3.673	1.543	0.000	1.779
Corchoionol C 9-glucoside	HMDB0029772	431. 192	3.674	4.472	0.000	1.724
Betulalbuside A	HMDB0035634	355. 173	4.278	3.904	0.000	1.246
Prenyl apiosyl-( 1− > 6)-glucoside	HMDB0031956	379. 161	3.546	1.250	0.000	1.196
(2E,4E,7R)-2,7-Dimethyl-2,4-octadiene- 1,8-diol 8-O-b-D-glucopyranoside	HMDB0038747	355. 173	3.901	7.401	0.000	1.187
[6]-Gingerdiol 5-O-beta-D-glucopyranoside	HMDB0036123	503.250	4.990	2.347	0.000	1.064
3,7-Dimethyl-5-octene- 1,7-diol 1-glucoside	HMDB0034771	357. 188	3.962	1.340	0.001	0.761
6-Feruloylglucose 2,3,4-trihydroxy-3-methylbutylglycoside	HMDB0036214	497. 163	4.363	1.441	0.002	− 0.443
Angelic acid	HMDB0029608	118.086	1. 184	1.483	0.000	− 2.146
Avenoleic acid	HMDB0029978	279.232	9.311	1.333	0.000	− 2.309
Mangiferic acid	HMDB0029800	263.237	8.565	1.810	0.000	− 2.355
5a,6a-Epoxy-7E-megastigmene-3b,9e-diol 9-glucoside	HMDB0038306	389.218	9.983	1.387	0.000	− 3.445
4,8,12,15-Octadecatetraenoic acid	HMDB0032672	277.216	9.709	1.684	0.000	− 3.581
Stearidonic acid	HMDB0006547	277.216	8.799	2.397	0.000	− 4.694
12-Hydroxy-8, 10-octadecadienoic acid	HMDB0029998	297.242	8.437	2.989	0.000	− 4.897
9, 10-DHOME	HMDB0004704	313.238	8.414	2.529	0.000	− 5. 173
(R)-1-O-[b-D-Glucopyranosyl-(1− > 6)-b-D-glucopyranoside]-1,3-octanediol	HMDB0032799	488.271	3.569	2.465	0.000	− 5.512
Corchorifatty acid F	HMDB0035919	309.207	8.814	1.747	0.000	− 5.921

### Correlation analysis of biological activities

The chemical composition of TPFD affects its efficacy. As mentioned above, 370 metabolites are significantly changed in the traditional processing of FSD into TPFD for TCM, the majority of which are organooxygen compounds, flavonoids, prenol lipids, fatty acids, and carboxylic acids and their derivatives. These metabolites have different activities and therefore may affect the efficacy of TPFD; therefore, future research should examine these 370 differentially abundant metabolites as potential chemical markers.

**Figure 5:**
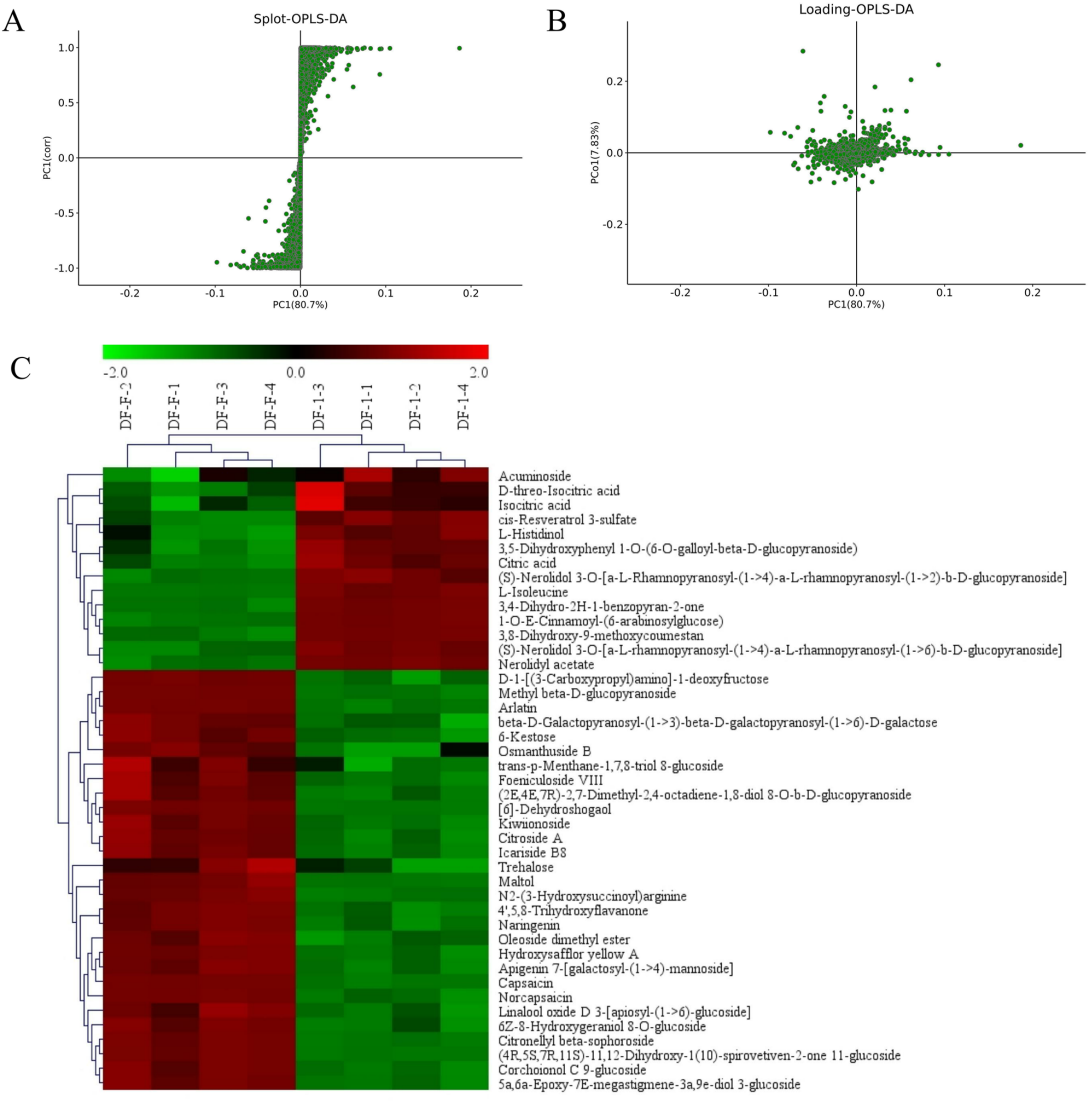
Orthogonal projections of latent structures discriminant analysis (OPLS-DA) and cluster analysis. (**A**): S-plots of the OPLS-DA model for the TPFD versus FSD sample comparison. (**B**): Loading of the OPLS-DA model for TPFD samples versus FSD samples. (**C**): Heatmap for TPFD samples versus FSD samples.

To provide visual evidence of the distinct nature of TPFD samples, the above OPLS-DA models were used to construct an S-plot and loading analysis (Fig. 5A and B), which provided a graphical projection of specific compounds. In these plots, metabolites close to the origin make a small contribution to the separation of the samples. A total of 43 metabolites (Fig. 5C) had a VIP score ≥ 4.0 in the OPLS-DA model, and a *t*-test revealed that they significantly differed (*P* < 0.05) between FSD and TPFD. In the S-plot and loading analysis, these compounds were farthest from the origin (in the positive and negative directions), indicating that they make a greater contribution to the distinction between samples. These 43 metabolites (listed in Table 6 with their activities) could therefore be used as chemical markers to assess whether the biological activity of *D. officinale* is altered through traditional processing.

**Table 6:** 43 biomarkers screened for TPFD vs FSD and their biological activities.

Metabolites	Compound ID	Class	VIP	log2(FC)	*P-value*	Activities	Reference
[6]-Dehydroshogaol	HMDB0033090	Cinnamic acids and derivatives	4.859	13.161	0.000	Anti-inflammatory Anti-oxidant	^34,35,36^
Capsaicin	HMDB0002227	Phenols	4.465	11.882	0.000	Anti-inflammatory;	^35^
						Analgesia	^37,38,39^
D-1-[(3-Carboxypropyl)amino]-1-deoxyfructose	HMDB0038663	Carboxylic acids and derivatives	8.636	11.310	0.000	N/A	N/A
Norcapsaicin	HMDB0036327	Phenols	4.355	9.189	0.000	Neuroprotection;	^40,41,42^
						Anti-Alzheimer.	
Arlatin	HMDB0035740	Prenol lipids	5.270	8.883	0.000	Anti-tumor	^43^
N2-(3-Hydroxysuccinoyl)arginine	HMDB0032765	Carboxylic acids and derivatives	4.284	5.518	0.000	Anti-inflammatory	^44^
(4R,5S,7R,11S)-11,12-Dihydroxy-1(10)-spirovetiven-2-one 11-glucoside	HMDB0033150	Prenol lipids	4.807	4.462	0.000	N/A	N/A
Maltol	HMDB0030776	Pyrans	7.266	4.351	0.001	Anti-bacterial,	^45^
						Anti-toxicity	^46^
4',5,8-Trihydroxyflavanone	HMDB0031824	Flavonoids	10.133	4.266	0.000	Anti-mutagenic	^47^
Naringenin	HMDB0002670	Flavonoids	5.399	4.219	0.000	Anti-bacterial,	^48^
						Anti-oxidant,	^49^
						Anti-tumor,	^50^
						Anti-inflammatory	^51^
Citronellyl beta-sophoroside	HMDB0032839	Prenol lipids	8.101	3.534	0.000	Anti-inflammatory	^52^
Methyl beta-D-glucopyranoside	HMDB0029965	Organooxygen compounds	5.375	3.525	0.000	Anti-tumor,	^53^
						Anti-bacterial,	^54^
						Anti-nociceptive,	^55^
						Anti-inflammatory	
Hydroxysafflor yellow A	HMDB0040677	Cinnamic acids and derivatives	6.967	3.302	0.000	Anti-bacterial,	^56^
						Anti-inflammatory	^57,58^
Apigenin 7-[galactosyl-(1->4)-mannoside]	HMDB0037852	Flavonoids	5.394	3.037	0.000	N/A	N/A
5a,6a-Epoxy-7E-megastigmene-3a,9e-diol 3-glucoside	HMDB0031676	Organooxygen compounds	4.134	2.426	0.000	N/A	N/A
Foeniculoside VIII	HMDB0033009	Organooxygen compounds	4.373	2.381	0.001	N/A	N/A
Kiwiionoside	HMDB0038691	Prenol lipids	4.578	2.217	0.000	N/A	N/A
Icariside B8	HMDB0036846	Prenol lipids	4.161	1.975	0.000	N/A	N/A
Corchoionol C 9-glucoside	HMDB0029772	Fatty Acyls	4.472	1.724	0.000	N/A	N/A
Citroside A	HMDB0030370	Prenol lipids	4.761	1.723	0.000	Hypertension,	^59^
						Anti-inflammatory	^60^
						Anti-oxidant and Anti-diabetic	^61^
6Z-8-Hydroxygeraniol 8-O-glucoside	HMDB0035025	Prenol lipids	5.028	1.480	0.000	N/A	N/A
beta-D-Galactopyranosyl-(1->3)-beta-D-galactopyranosyl-(1->6)-D-galactose	HMDB0038853	Organooxygen compounds	5.111	1.441	0.000	N/A	N/A
Oleoside dimethyl ester	HMDB0031350	Prenol lipids	4.651	1.346	0.000	Anti-oxidant	^62^
							^63^
6-Kestose	HMDB0033673	Organooxygen compounds	4.641	1.289	0.000	N/A	N/A
(2E,4E,7R)-2,7-Dimethyl-2,4-octadiene-1,8-diol 8-O-b-D-glucopyranoside	HMDB0038747	Fatty Acyls	7.401	1.187	0.000	N/A	N/A
Linalool oxide D 3-[apiosyl-(1->6)-glucoside]	HMDB0031367	Organooxygen compounds	4.502	0.881	0.000	N/A	N/A
trans-p-Menthane-1,7,8-triol 8-glucoside	HMDB0034784	Organooxygen compounds	4.657	0.628	0.003	N/A	N/A
Osmanthuside B	HMDB0038749	Cinnamic acids and derivatives	4.202	0.567	0.001	Anti-depressant,	^64^
						Inflammatory,	^65^
						Anti-oxidant,	^66,67^
						Lipase inhibition	
Trehalose	HMDB0000975	Organooxygen compounds	7.692	0.301	0.004	Anti-depressant	^68^
Acuminoside	HMDB0029347	Prenol lipids	4.358	-0.294	0.031	Immune enhancer abd Anti-inflammatory	^69^
Isocitric acid	HMDB0000193	Carboxylic acids and derivatives	4.588	-0.548	0.017	Surfactants, detergents, ion chelators and biologically active,	^70^
						Anti-oxidant	^71,72^
L-Histidinol	HMDB0003431	Organonitrogen compounds	6.333	-0.585	0.000	Against CDDP, Nephrotoxicity, cytoprotective and attenuate fanconi syndrome, Anti-tumour activity and cardiotoxicity	^73^ ^74^ ^75^
3,5-Dihydroxyphenyl 1-O-(6-O-galloyl-beta-D-glucopyranoside)	HMDB0039307	Organooxygen compounds	8.940	-0.709	0.000	Anti-oxidant	^76^
D-threo-Isocitric acid	HMDB0001874	Carboxylic acids and derivatives	4.778	-0.831	0.001	Anti-inflammatory	^77^
cis-Resveratrol 3-sulfate	HMDB0041712	Stilbenes	7.225	-0.906	0.000	N/A	N/A
Citric acid	HMDB0000094	Carboxylic acids and derivatives	8.410	-1.718	0.000	Anti-oxidant, Anti-fatigue	^78^ ^79^
(S)-Nerolidol 3-O-[a-L-Rhamnopyranosyl-(1->4)-a-L-rhamnopyranosyl-(1->2)-b-D-glucopyranoside]	HMDB0040845	Organooxygen compounds	5.223	-1.917	0.000	N/A	N/A
(S)-Nerolidol 3-O-[a-L-rhamnopyranosyl-(1->4)-a-L-rhamnopyranosyl-(1->6)-b-D-glucopyranoside]	HMDB0040846	Organooxygen compounds	6.236	-2.019	0.000	N/A	N/A
3,4-Dihydro-2H-1-benzopyran-2-one	HMDB0036626	3,4-dihydrocoumarins	8.459	-2.892	0.000	Anti-tyrosinase	^80^
L-Isoleucine	HMDB0000172	Carboxylic acids and derivatives	8.430	-2.970	0.000	Anti-oxidant	^81^
1-O-E-Cinnamoyl-(6-arabinosylglucose)	HMDB0030294	Cinnamic acids and derivatives	11.458	-3.554	0.000	N/A	N/A
3,8-Dihydroxy-9-methoxycoumestan	HMDB0030562	Isoflavonoids	4.619	-4.279	0.000	N/A	N/A
Nerolidyl acetate	HMDB0039630	Prenol lipids	4.241	-7.130	0.000	Anti-inflammatory	^82^

Of the 43 chemical markers, 29 were more abundant in TPFD than in FSD samples, while 14 were less abundant. Their medicinal properties include anti-inflammatory, anti-mutagenic, analgesic, neuroprotection and anti-Alzheimer’s, anti-tumor, antibacterial, anti-toxicity, antioxidant, anti-nociceptive, anti-hypertension, anti-diabetic, anti-depressant, lipase-inhibiting, immune-enhancing, cis-diaminedichloroplatinum nephrotoxicity-preventing, cytoprotective, Fanconi syndrome–attenuating, cardiotoxicity-preventing, anti-fatigue, and anti-tyrosinase activities. Anti-inflammatory activity is the most common function of the significantly upregulated metabolites (Table 6). Among the 43 chemical markers, [6]-dehydroshogaol and capsaicin (Fig. 4) showed the greatest difference in abundance between TPFD and FSD samples, with log2 fold-change values of 13.16 and 11.88, respectively. Imm et al. established that capsaicin and [6]-dehydroshogaol inhibited the production of nitric oxide (NO) in LPS-stimulated cells in a dose-dependent manner^34^. These chemicals are also likely to have anti-inflammatory and antioxidant effects by inactivating the eukaryotic transcription factor NF-kB^35,36^. Furthermore, the log2 fold-change values of the anti-inflammatory compounds N2-(3-hydroxysuccinoyl)arginine, naringenin, citronellyl beta-sophoroside, methyl beta-D-glucopyranoside, and hydroxysafflor yellow A (Fig. 4) were > 3 (Table 6). Thus, TPFD has better anti-inflammatory properties than FSD, which is beneficial for its applications in TCM.

In addition, the 43 marker metabolites included compounds with anti-tumor and anti-diabetic activities. Arlatin, naringenin, and methyl beta-D-glucopyranoside (Fig. 4) showed significant anti-tumor activity, while citroside A (Fig. 4) possesses significant anti-diabetic activity. The contents of all these compounds were significantly higher in TPFD than in FSD samples. These results are consistent with the reported effects of TPFD in TCM, providing scientific evidence of the efficacy of the traditional application of *D. officinale*.

We also detected some compounds in *D. officinale* with potential therapeutic effects on neurological diseases; for example, norcapsaicin (Fig. 4) has neuroprotection and anti-Alzheimer’s activities, while osmanthuside B (Fig. 4) shows anti-depressant activity. Future studies should explore the relationship between the concentrations of these compounds present and the therapeutic effects and health-promoting properties of *D. officinale* products.

## Discussion

### The significant differences on metabolites between FSD and TPFD

We integrated UHPLC coupled with Q Exactive plus quadrupole-Orbitrap MS in the positive and negative ion modes, combined with a HMDB and multivariate statistical analysis for qualitative analyses, to screen the different constituents of FSD and TPFD. The result of this study reveals that five type compounds including organooxygen compounds, prenol lipids, flavonoids, carboxylic acids and their derivatives, and fatty acyls show the significant differences among 370 differential metabolites which were unambiguously detected or tentatively identified. FSD and TPFD samples contained all the identified metabolites, there is a large amount of metabolites data available on FSD and TPFD under different processing. But the relative contents of individual compounds were remarkably different between the two groups.

### The possible mechanism of substance and its bioactivities

Chemical structures of typical compounds and related bioactive in TPFD have been identified by OPLS-DA methods, For example, 43 metabolites were identified as chemical markers that could be used to distinguish FSD and TPFD samples according to qualitative research, and the contents of some compounds with anti-inflammatory and antioxidant, such as [6]-dehydroshogaol, capsaicin, arlatin, naringenin, methyl beta-D-glucopyrano-side, and citroside A, significantly increased after processing according to quantitative research. This finding shows that traditional processing could possibly changed the contents of partial active compounds and improved the efficiency of *D. officinale*.

### Anti-inflammatory and antioxidant related substance bases

**Figure 6:**
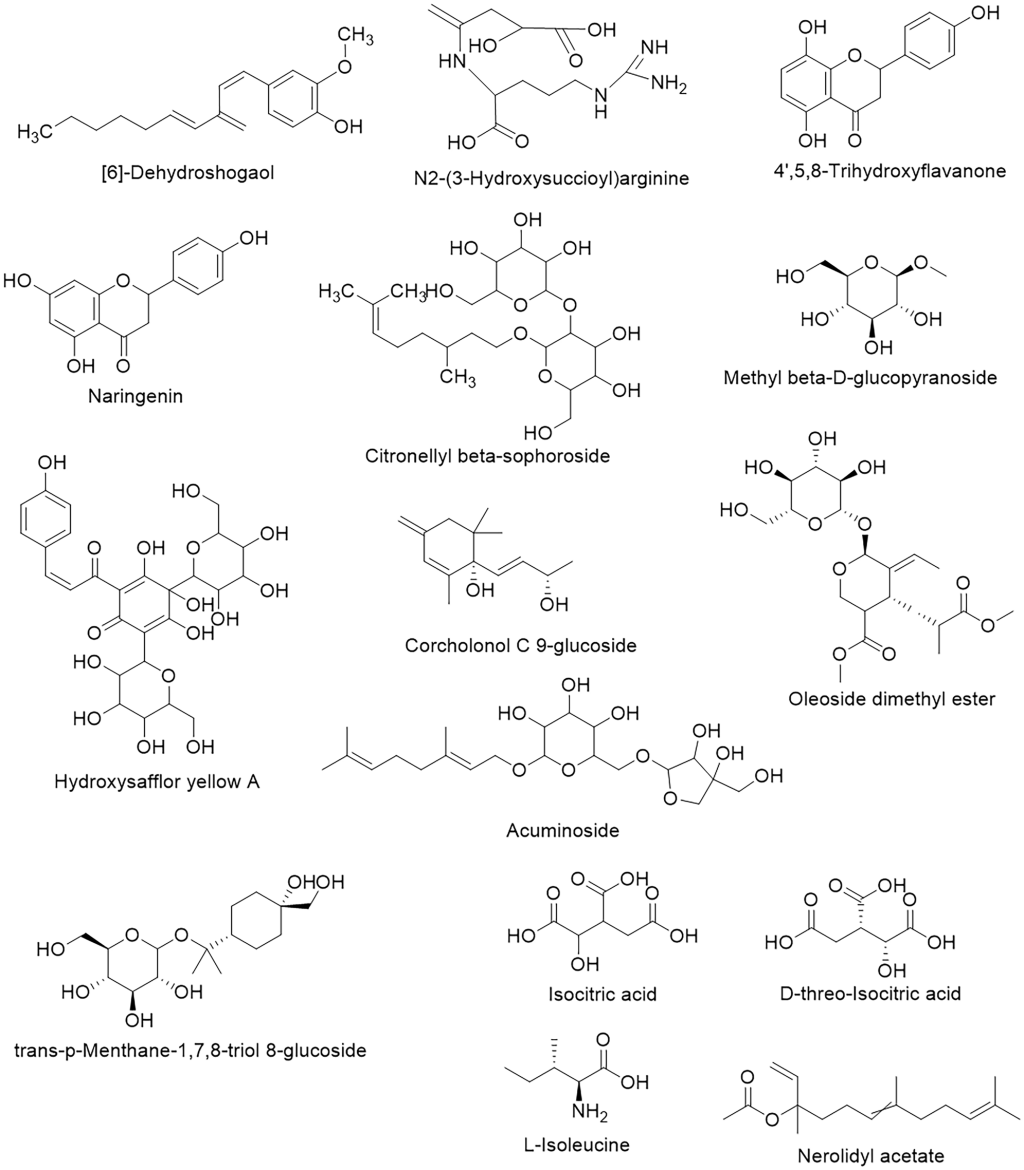
Chemical constructure of 15 significant increased compounds.

15 of 43 biomarkers screened for TPFD versus FSD, including [6]-Dehydroshogaol, N2-(3-Hydroxysuccinoyl)arginine, 4',5,8-Trihydroxyflavanone, Naringenin,Citronellyl beta-sophoroside, Methyl beta-D-glucopyranoside, Hydroxysafflor yellow A, Corchoionol C 9-glucoside, Oleoside dimethyl ester, trans-p-Menthane-1,7,8-triol 8-glucoside, Acuminoside, Isocitric acid, D-threo-Isocitric acid, L-Isoleucine, Nerolidyl acetate, presented anti-inflammatory and antioxidant bio-activities. Their chemical constructure show in following (Fig. 6).

This study found that 40 flavonoids were significantly different in TPFD and FSD samples, of which 26 flavonoids more abundant in TPFD than in FSD samples, while 14 flavonoids were significantly less abundant. Flavonoids as anti-inflammatory and antioxidant substances, two key substances, Naringenin and 4',5,8-Trihydroxyflavanone of flavonoids’ compounds, have outstanding changed (Table 3) so that TPSD can better exert its anti-inflammatory and antioxidant effects (Table 6).

In order to promote further research into FSD and TPFD, attentions should be paid to the following work in the future: (1) Although current metabolites technologies have been used to study TPFD, there are limitedly comprehensive metabonomics studies. Obviously, the negative or positive ion model method such as metabolomics cannot satisfy the necessary deep research into *D. officinale*^21^. (2) it is important to comprehensively investigate why differences exist in active compounds between FSD and TPFD which can provide propitiate conditions for metabolite accumulation. (3) its TPFD applications has been rarely described, and TPFD improvements are still required for its industrial applications. (4) the functions of most these compositions need to confirm through functional investigation.

## Conclusion

For thousands of years, *D. officinale* has been processed to enhance its medicinal value for use in TCM. The chemical composition of this material after processing is key to its efficacy. Traditional processing of *D. officinale* produces a difference in the contents of key metabolites. Moreover, combining metabolomics and multivariate statistical analysis methods can accurately identify markers that differentiate processed and raw materials. Thus, we revealed the basis of the improvement in efficacy of TPFD compared with FSD, enabling the identification of active substances with functions not included in the traditional use of TPFD. These results indicate the need for the further exploration of TPFD in the treatment of additional, previously untested conditions. This systematic study of FSD and TPFD provides a useful analytical strategy for rapidly screening and identifying the constituents of other TCMs and TCM formulas. In addition, the results of this research provide a theoretical basis for quality control.

## Abbreviations

TPFD: Tipifengdou
FSD: Fresh stems of *D. officinale*
TCM: Traditional Chinese medicines
UHPLC-QEQO/MS: Ultra-high-performance liquid chromatography coupled with Q-Exactive plus quadrupole-Orbitrap mass spectrometry
HMDB: Human Metabolome Database
PCA: Principal component analysis
OPLS-DA: Orthogonal projections to latent structures discriminant analysis
PLS-DA: Partial least squares discriminant analysis
VIP: Variable importance of projection
ESI: Electrospray ionization
TIC: Typical total ions current chromatogram
NF-kB: Nuclear factor-k-gene binding
