# Author Correction: Traditional processing increases biological activities of *Dendrobium offificinale* Kimura et. Migo in Southeast Yunnan, China

**DOI:** 10.1038/s41598-023-30276-w

**Published:** 2023-02-23

**Authors:** Di Zhou, Ying Zhao, Zhilin Chen, Xiuxiang Yan, Yanqiang Zhao, Lu Gao, Lixin Yang

**Affiliations:** 1 Key Laboratory of Economic Plants and Biotechnology, Kunming Institute of Botany, Chinese Academy of Sciences, Kunming, 650201 Yunnan China; 2 Bio‑Innovation Center of DR PLANT, Kunming Institute of Botany, Chinese Academy of Sciences, Kunming, 650201 Yunnan China; 3 Center of Biodiversity and Indigenous Knowledge, Kunming, 650034 Yunnan China; 4 College of Forestry and Vocational Technology in Yunnan, Kunming, 650224 Yunnan China; 5 School of Ethnic Medicine, Yunnan Minzu University, Kunming, 650504 Yunnan China; 6 College of Pharmacy, Guizhou University of Traditional Chinese Medicine, Guiyang, 550025 Guizhou China

Correction to: *Scientific Reports* 10.1038/s41598-022-17628-8, published online 31 August 2022

The original version of this Article contained errors.

In Table 6, Reference 62 was incorrectly given as Reference 82.

The correct Reference 62 is listed below:

He, Z.D. et al. Antioxidative glucosides from the fruits of Ligustrum lucidum. Chem. Pharm. Bull. 49, 780–784 (2001).

As a result, the subsequent References have been renumbered.

In addition, in the Acknowledgements section,

“The work also supported Southeast Asia Biodiversity Research Institute, Chinese Academy of Sciences (2015CASEABRIRG001).”

now reads:

“This work was also supported by Southeast Asia Biodiversity Research Institute, Chinese Academy of Sciences (Grant Nos. 2015CASE-ABRIRG001 and Y4ZK111B01).”

The original Article has been corrected.

